# Advances in biomaterials as a retinal patch for the repair of rhegmatogenous retinal detachment

**DOI:** 10.3389/fbioe.2022.997243

**Published:** 2022-10-17

**Authors:** Chuanzhen Zheng, Dejia Wen, Kejia Xu, Xiaomin Zhang, Xinjun Ren, Xiaorong Li

**Affiliations:** ^1^ Tianjin Key Laboratory of Retinal Functions and Diseases, Tianjin International Joint Research and Development Centre of Ophthalmology and Vision Science, Eye Institute and School of Optometry, Tianjin Medical University Eye Hospital, Tianjin, China; ^2^ Tianjin Fourth Central Hospital, Department of Ophthalmology, Tianjin, China

**Keywords:** rhegmatogenous retinal detachment, biomaterials, biocompatibility, categories, synthetic material, polyethylene glycol

## Abstract

Rhegmatogenous retinal detachment (RRD) is the most common retinological emergency that can cause blindness without surgical treatment. RRD occurs when liquefied vitreous accumulates between the neurosensory retina and the retinal pigment epithelium *via* retinal breaks, which are caused by the separation of the vitreous from the retina with aging. Currently, the main treatment option is pars plana vitrectomy, which involves surgical removal of the vitreous and laser photocoagulation around retinal breaks to generate firm chorioretinal adhesion, as well as subsequent filling of the vitreous cavity with long-lasting substitutes (expansile gas or silocone oil) to prevent the connection between the subretinal space and the vitreous cavity *via* the breaks before the chorioretinal adhesion firm enough. However, the postoperative face-down position and the not very satisfactory first retinal reattachment rate place a heavy burden on patients. With the development of technology and materials engineering, researchers have developed biomaterials that can be used as a retinal patch to seal retinal breaks and prevent the connection of subretinal space and vitreous cavity *via* breaks, thus replacing the long-lasting vitreous substitutes and eliminating the postoperative face-down position. Preclinical studies have demonstrated that biomaterial sealants have enough biocompatibility and efficacy in the *in vitro* and *in vivo* experiments. Some sealants have been used in clinical trials on a small scale, and the results indicate promising application prospects of the biomaterial sealants as retinal patches in the repair of RRD. Herein, we review the recent advances in biomaterials as retinal patches for the repair of RRD, focusing on the biomaterial categories, methods, and procedures for sealing retinal breaks, as well as their biocompatibility and efficacy, current limitations, and development perspectives.

## Introduction

**FIGURE 1:**
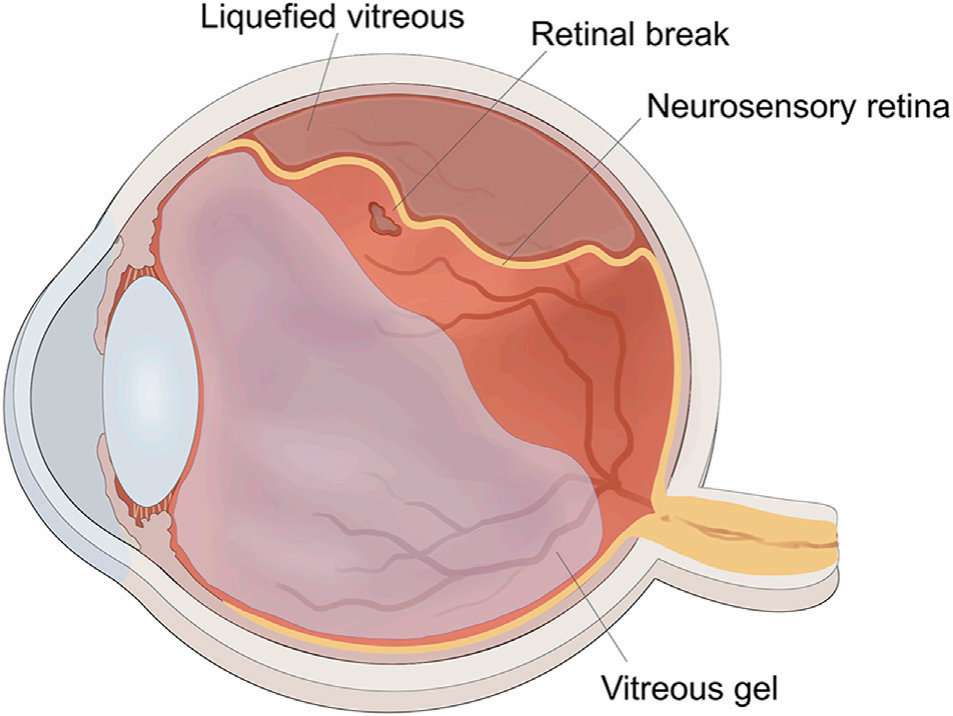
The schematic diagram of rhegmatogenous retinal detachment. The traction of vitreous gel onto retina created the retinal break, liquified vitreous penetrated into the subretinal space through the retinal break to induce retinal detachment.

**FIGURE 2:**
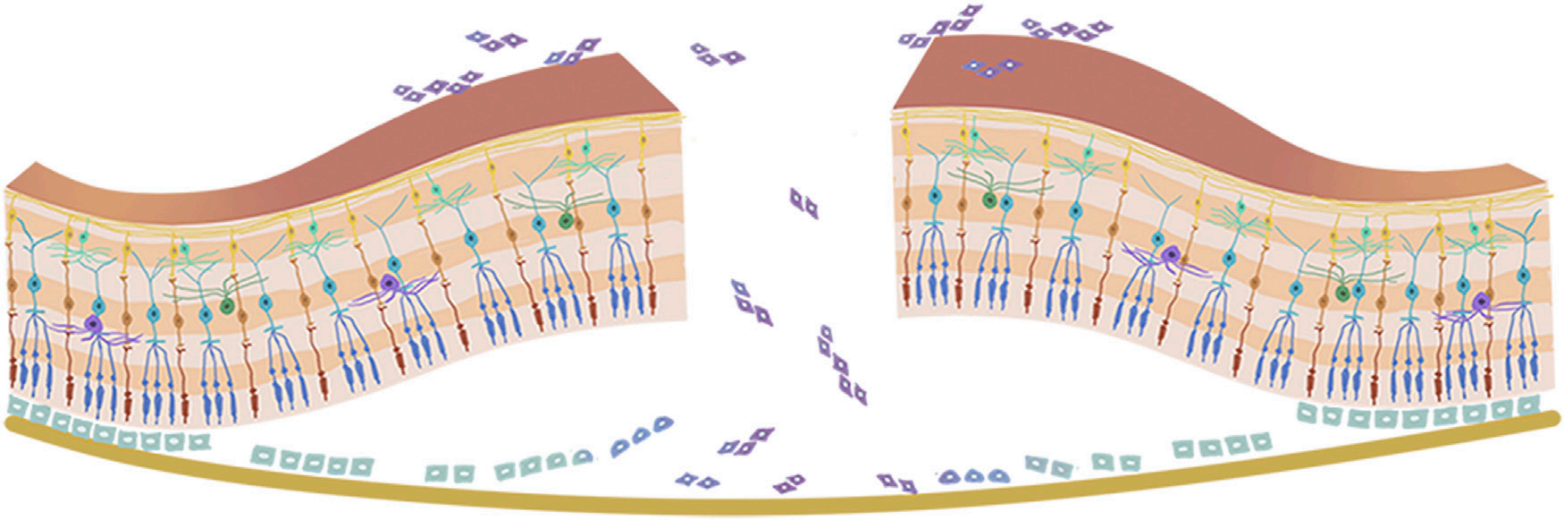
Pathophysiology of proliferative vitreoretinopathy. The retinal pigment epithelium cells lose cell-cell contact, and diffuse into the vitreous cavity *via* retinal breaks, then go through epithelial-mesenchymal transition (EMT) and eventually transform into myofibroblasts.

The vitreous body is situated between the lens and retina, and accounts for approximately 80% of the volume of the eye globe. It aids in keeping the retina in place and provides nourishment to the eye. The vitreous humor is a transparent gel-like substance that is predominantly composed of water (98–99%), long fine collagen fibers, and hyaluronic acid (HA). Aging leads to homogeneous vitreous liquefaction into a heterogeneous mixture, including aggregated collagen fibrils and dissociated hyaluronans, because of various factors such as oxidative damage, digestion by enzymes, and collagen mutations. (Tram and Swindle-Reilly, 2018) As the gel network collapses, the vitreous separates from the retina, which may induce retinal breaks at the site if the forces of separation are significantly strong or if there is abnormal adhesion between the vitreous gel and the retina. The flow of liquefied vitreous into the subretinal space *via* these retinal breaks held open by vitreoretinal traction leads to separation of the neurosensory retina from the underlying retinal pigment epithelium (RPE), that is, rhegmatogenous retinal detachment (RRD) (Figure 1). (Feltgen and Walter, 2014; Lumi et al., 2015) In the pathological process of RRD, not only does the liquefied vitreous enter the subretina through retinal breaks, but the RPE cells also lose cell-cell contact and diffuse into the vitreous cavity *via* retinal breaks, undergo epithelial-mesenchymal transition (EMT), and eventually transform into myofibroblasts. This causes the development of proliferative fibrocellular membranes with the ability to contract, which facilitates the progression of tractional retinal detachment (Figure 2). (Pastor et al., 2016; Zheng et al., 2022).

RRD is one of the most common retinological crises, and must be treated as quickly as possible. (Yorston et al., 2021) In view of the fact that the three necessary pre-requisites for the progression of RRD are the flow of liquefied vitreous, vitreoretinal tractional forces, and retinal breaks, the management principles are to treat all the retinal breaks and weaken or eliminate vitreoretinal traction to prevent the penetration of fluid into the subretinal space *via* retinal breaks. Invasive procedures include single pneumatic retinopexy (PR), scleral buckling (SB), and pars plana vitrectomy (PPV), as well as any combination of these. (Kuhn and Aylward, 2014) PR is the intravitreal injection of an expanding gas bubble to act as a tamponade to re-attach the retina. Although this technique is simple and causes minimal damage, its indications are limited. It has only been recommended for individuals with small, anterior, superior retinal breaks and little or no proliferative vitreoretinopathy (PVR). (Kunikata et al., 2019; Chronopoulos et al., 2021) SB is performed after accurate localization of all retinal breaks and precise cryotherapy around retinal breaks for scar induction, and by suturing foam sponges or silicone bands to the sclera to cause an inward indentation of the sclera and choroid to neutralize the traction of the vitreous on the retina. (Kuhn and Aylward, 2014; Kunikata et al., 2019) PPV begins with a thorough removal of the vitreous, which eliminates vitreoretinal tractional force and resets the detached retina, followed by laser photocoagulation around the edge of the retinal breaks to generate chorioretinal adhesion, and injection of the vitreous cavity with long-acting alternatives (expansile gas or silocone oil) to act as a tamponade. The fundamental role of long-lasting vitreous substitutes is to keep the neurosensory retina attached to the RPE and prevent liquid flow between the vitreous cavity and the subretinal space until the chorioretinal adhesion becomes firm enough to seal the retinal breaks. (Feltgen and Walter, 2014; Zheng et al., 2022) However, the tamponade property of long-lasting vitreous substitutes requires a long-term postoperative face-down position, given that their gravity is lower than that of the aqueous humor. Moreover, the visual quality is poor during this period because the refractive index of vitreous substitutes is lower or higher (1.00 in expansile gas and 1.40 in silocone oil) than in the natural human vitreous (1.33). (Hoshi et al., 2019) The refractive status returns to normal after the expansile gas is absorbed within 2 weeks to 2 months, or after the silicone oil is removed *via* a second surgical procedure, because the refractive index of the aqueous fluid produced by the eye itself and filled in the vitreous cavity is identical to that of the natural vitreous. (Wagenfeld et al., 2010; Chen et al., 2015; Kontos et al., 2017; Raczynska et al., 2018; Tetsumoto et al., 2020; Popovic et al., 2022) For simple RRD with anterior retinal breaks, there were no significant differences between RRD and SB in terms of the primary success rate, visual acuity gain, and final anatomical success. (Znaor et al., 2019) However, for some more challenging situations, such as giant retinal breaks, multiple large breaks, bullous detachment, and cases complicated by severe PVR, isolated PPV or PPV and SB combined with long-lasting vitreous substitutes are the first-line treatment options. (Wang and Snead, 2020).

With evident medical and technical breakthroughs, PPV has increased in popularity and has become the most commonly performed surgery for the treatment of RRD, albeit with an initial retinal reattachment rate ranging from 74% to 96.3%. (Minihan et al., 2001; Bourla et al., 2010; Oshima et al., 2010; Kobashi et al., 2014; Haugstad et al., 2017; Romano et al., 2017) The most prevalent cause of surgical failure in retinal detachment is PVR. RPE cells are crucial contributors in triggering PVR. Therefore, it would make sense to prevent PVR by inhibiting RPE cell migration, proliferation, and EMT through retinal breaks by patching the breaks with an appropriate substance. Likewise, the application of such a material could effectively block communication between the two sides of the breaks, thereby eliminating the need for long-lasting vitreous substitutes and the postoperative face-down position. This review summarizes the materials, methods, and procedures for patching retinal breaks as well as their biocompatibility and efficacy in the repair of RRD, which could provide basic and comprehensive information.

## Clinical biomaterials

**FIGURE 3:**
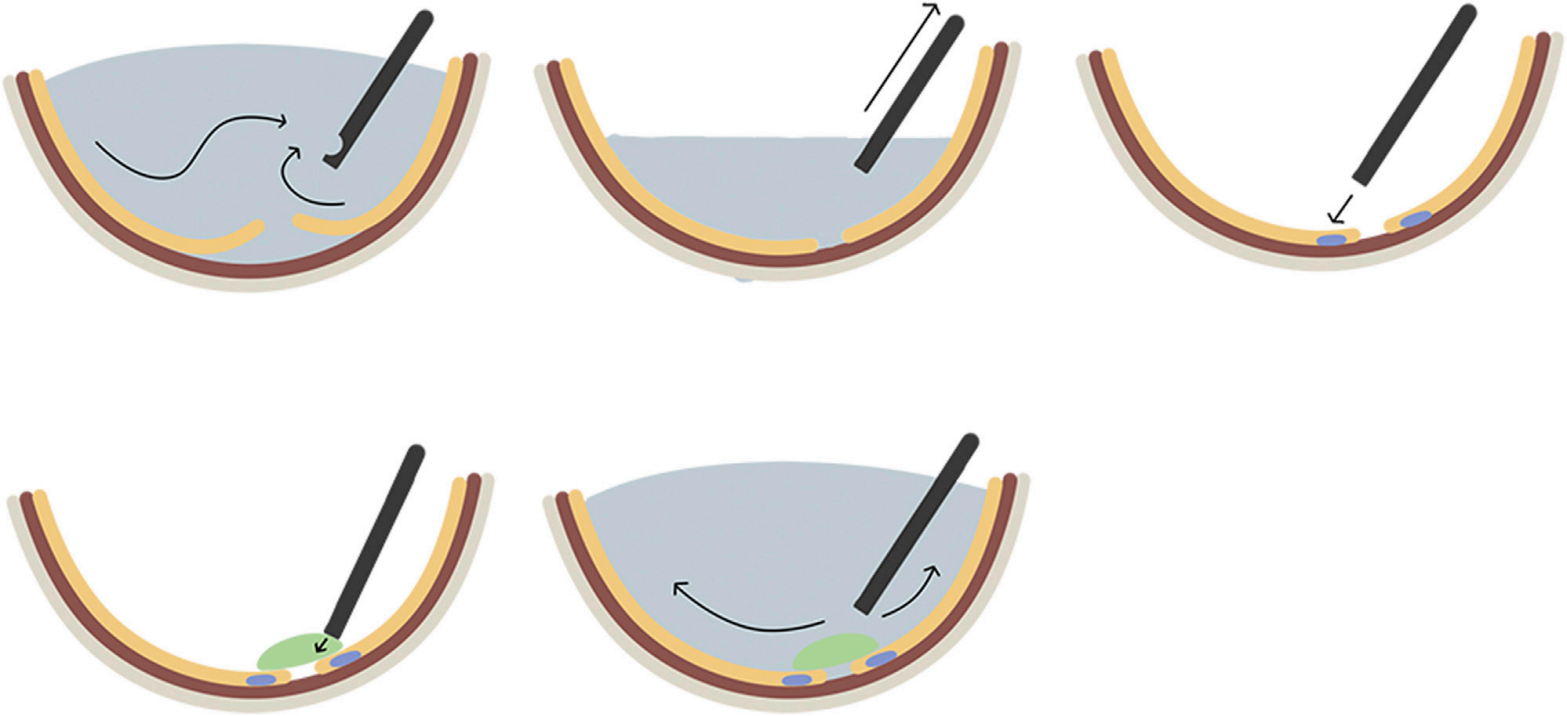
The general surgical steps of applying biomaterials as retinal patches for the repair of rhegmatogenous retinal detachment. A. complete pars plana vitrectomy; B. fluid-air exchange to remove subretinal fluid and achieve retinal reattachment; C. laser photocoagulation around retinal break; D. full coverage of retinal breaks with biomaterial sealants; E, fluid-air exchange again to fill the vitreous cavity with the balanced salt solution.

Surgical glues are useful adjuncts to surgical care and are classified as hemostats, adhesives, and sealants, based on their function. Hemostats act as hemostatic agents to clot the blood. While adhesives bond two surfaces together, like wounds or incisions, sealants mostly develop a barrier layer to prevent fluid or air leakage. It is noteworthy that the same glue can perform numerous functions. For instance, fibrin glue can function as a sealant, adhesive, or hemostat. (Jain and Wairkar, 2019) A retinal sealant is utilized in the vitrectomy repair of RRD to build a seal over the retinal breaks before the chorioretinal adhesion becomes firm enough to avoid the migration of RPE into the vitreous, thus reducing the subsequent PVR. The general surgical steps are complete PPV followed by fluid-air exchange to remove subretinal fluid and achieve retinal reattachment, laser photocoagulation around retinal breaks under air conditions, full coverage of retinal breaks with retinal sealants, and finally leaving sterile air to fill the vitreous cavity or proceeding with fluid-air exchange again to allow balanced salt solution (BSS) to fill the vitreous cavity (Figure 3). An ideal retinal sealant must have certain characteristics to create a stable and safe seal around the retinal breaks: (A) Biocompatibility indicates that it does not cause local irritation, inflammation, toxicity, or antigenicity. (B) Strong and stable persistent adhesion means that it must remain firmly attached and adherent to the retina for an appropriate time frame to permit the establishment of chorioretinal adhesion induced by laser photocoagulation. (C) Owing to its flexibility, it should be delivered to the target surface *via* a simple procedure. (D) Biodegradability: It should be naturally absorbable to avoid the long-term negative effects of persistent foreign bodies and the need for a second surgery to remove it.

Currently, there are two major categories of tissue sealants used in ophthalmic surgery: natural compound-based sealants (such as protein derivatives and polysaccharide derivatives) and synthetic material-based sealants [such as cyanoacrylate and polyethylene glycol (PEG) derivatives]. (Guhan et al., 2018; Jain and Wairkar, 2019) Sealants derived from natural biopolymers are obtained from biological constituents, which provide many benefits over synthetic materials, such as greater biocompatibility, lower immunological response, *in vivo* degradability, and the potential to guide and promote endogenous reparative processes. Conversely, sealants derived from synthetic polymeric materials also offer some advantages over natural biomaterials because of their well-defined chemical compositions, such as reproducibility, more precise control over gel time, swelling behavior, mechanical properties, adjustable mechanical strength, degradation rate, and byproducts. These advantages make it possible to tailor the materials for specific biomedical and biotechnological applications. (Heher et al., 2018) Table 1 summarizes the studies on different sealants for RRD surgery.

**TABLE 1:** The summaries of different sealants as retinal patches for RRD surgery applications.

Origin of tissue sealants	Sealant class	Cross-linking or polymerization	Research progress
Sealants of natural origin	Fibrin glue	Enzymatic crosslinking	Clinical trails
	Gelatin-based sealants	Enzymatic crosslinking or photocrosslinking	Preclinical studies
	Seprafilm	Chemical crosslinking	Clinical trails
	Healaflow	Chemical crosslinking	Clinical trails
	dispersive OVD	Chemical crosslinking	Preclinical studies
Sealants of synthetic materials	Cyanoacrylate	Chemical crosslinking	Animal experiments have confirmed retinal toxicity, no further exploration.
	PEG-based sealants	Chemical or photochemical crosslinking	Preclinical studies

OVD, Ophthalmic viscosurgical devices; PEG, polyethylene glycol.

## Sealants of natural origin

Sealants of natural origin are classified into two types: those based on proteins (such as fibrin and gelatin-based sealants) and those based on polysaccharides (such as HA-based and chondroitin sulfate-based sealants). Nevertheless, the use of original natural sealants is hindered by some limitations, such as poor mechanical properties, weak manipulability, and rapid degradation. Cross-linking can compensate for these undesirable deficiencies, while maintaining the biocompatibility and biodegradability of the original materials. Cross-linking is a polymer stabilization procedure that results in multidimensional extension of polymer chains by attaching one polymer to another, consequently forming a stable network structure. (Poudel et al., 2022) Sealants are divided into two categories according to the type of cross-linking junctions: physically and chemically cross-linked. Physically cross-linked networks have transient junctions between polymeric chains that are mediated by chain entanglements or physical interactions such as hydrophobic interactions, hydrogen bonding, or ionic interactions. Chemically cross-linked networks have permanent junctions, which refer to the intermolecular or intramolecular joining of two or more molecules by a covalent bond *via* the introduced cross-linkers. (Arora et al., 2017; Lu et al., 2018) As no extra chemical components are added to the physically cross-linked networks, they ensure the favorable biocompatibility of the original materials. However, some added cross-linkers in chemically cross-linked networks may cause cytotoxicity that the human body cannot endure at high concentrations. (Jeon et al., 2007; Lai, 2014).

Protein-based materials are more often employed, whereas polysaccharide-based systems are very popular and desirable. The representative sealants are described below. Table 2 and Table 3 summarizes these two different materials as retinal patches for RRD surgery.

**TABLE 2:** The summaries of studies about sealants based on proteins as retinal patches for the treatment of RRD.

Sealants	Brand/Component	Authors	Date	Preclinical studies	Clinical trails
Fibrin glue	TISSEEL Kit, Baxter AG, Vienna, Austria	Mudit et al. (Tyagi and Basu, 2019)	2019	—	Fibrin glue replaced the long-lasting vitreous substitutes, and reached 100% retinal reattachment (5 cases).
	TISSEEL Kit, Baxter AG, Vienna, Austria	Erdinc et al. (Aydin et al., 2021)	2021	—	Fibrin glue replaced the laser photocoagulation around retinal breaks, and reached 100% retinal reattachment (5 cases).
	Bei Xiu Biotech Co. Ltd., Guangzhou, China	Wang et al. (Wang et al., 2020)	2020	—	Fibrin glue replaced the long-lasting vitreous substitutes, and reached 100% retinal reattachment (26 cases).
Gelatin-based sealants	gelatin-mTG	Chen et al. (Chen et al., 2006)	2006	*In vitro* study about adhesive strength.	None
				*In vivo* study about biocompatibility of two component in rat eyes.	
	gelatin-mTG	Yamamoto et al. (Yamamoto et al., 2013)	2013	*In vivo* study about biocompatibility and efficacy in RRD model of rabbit eyes.	None
	GelMA	Chen et al. (Chen et al., 2021)	2021	*In vitro* study about physicochemical properties of GelMA with different MA substitutes and concentrations.	None
				*In vivo* study about biocompatibility in rabbit eyes.	

**TABLE 3:** The summaries of studies about sealants based on polysaccharides as retinal patches for the treatment of RRD.

Sealants	Authors	Date	Preclinical studies	Clinical trails
Seprafilm	Jun et al. (Sueda et al., 2006)	2006	*In vitro* study about physicochemical properties.	—
			*In vivo* study about biocompatibility.	
	Teruya et al. (Teruya et al., 2009)	2009	*In vivo* study about biocompatibility and efficacy in RRD model of rabbit eyes.	—
	Haruta et al. (Haruta et al., 2017)	2017	—	Seprafilm replaced the long-lasting vitreous substitutes, achieving 100% (4 cases) retinal reattachment and confirming long-term safety (9 years follow-up).
Healflow	Barth et al. (Barth et al., 2014)	2014	*In vitro* study about biocompatibility.	—
	Barth et al. (Barth et al., 2016)	2016	*In vivo* study about biocompatibility.	—
	Barth et al. (Barth et al., 2019)	2019	*In vivo* study about biocompatibility.	—
	Ren et al. (Ren et al., 2020)	2020	*In vitro* study about adhesion and duration in BSS.	Healaflow replaced the long-lasting vitreous substitutes, and achieved 97.4% retinal reattachment (37 eyes) at first, after the second surgery in one eye due to failure of the chorioretinal scar around retinal breaks, 100% reattachment rate was achieved.
Dispersive OVD	Hirata et al. (Hirata et al., 2013)	2013	*In vivo* study about biocompatibility and efficacy in RRD model of rabbit eyes.	None

### Sealants based on protein

#### Fibrin glue

**FIGURE 4:**
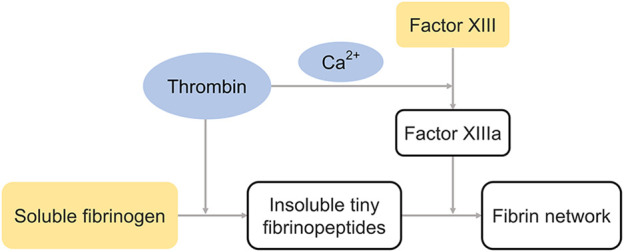
The cross-linking process of fibrin glue. Thrombin catalyzes the formation of insoluble tiny fibrinopeptides and factor XIIIa, and factor XIIIa catalyzes the transformation of fibrinopeptides to fibrin network. The blue and yellow represent components in different syringes.

Fibrin is a protein found in the human blood that assists in blood clotting. Fibrin glue is a biological sealant derived from blood products, and its working principle is to imitate the final stages of the coagulation cascade, wherein the fibrin clot is formed. Fibrin glue consists of two components drawn in separate syringes. The first is primarily composed of human plasma proteins, including fibrinogen, factor XIII, fibronectin, and plasminogens. The second is the combination of thrombin and calcium chloride. The transformation of fibrinogen to fibrin glue after the mixture of the two solutions consists of three main steps: 1) thrombin cleaves soluble fibrinogen into insoluble tiny fibrinopeptides; 2) thrombin also stimulates factor XIII to form transglutaminase-factor XIIIa in the presence of calcium ions; and 3) factor XIIIa catalyzes the transformation of fibrinopeptides to fibrin network *via* the formation of amide bonds between the lysine (Lys) and glutamine (Gln) residues (Figure 4). Cross-linking contributes to the stability and deformation resistance of fibrin clots as well as resistance to solubilization. Hence, transglutaminases are recognized as natural biological crosslinkers. The conversion of the solution to a milky white color can be observed when the glue begins to gel. Moreover, the concentration of the thrombin activation solution determines the formation rate of fibrin glue, while the adhesive strength of fibrin glue is directly proportional to the concentration of available fibrinogen. (Brennan, 1991; Scognamiglio et al., 2016) Finally, fibrin glue is progressively absorbed by a biological process in which the tissue plasminogen activator (tPA) cleaves plasminogen into plasmin, which subsequently lyses fibrin into soluble fibrin degradation products. (Brennan, 1991).

With the advancement of technology, biologically derived fibrin glue can now be mass-produced with meticulousness to eliminate any possibility of contamination or inclusion of infectious organisms. The sealing properties of fibrin glue have been extensively described in the fields of pulmonary and anorectal surgery, where it is particularly effective in preventing alveolar air leakage and closing fistula tracts. (Zmora et al., 2003; Kawamura et al., 2005) Furthermore, fibrin glue has been shown to be safe and effective as an ocular tissue sealant in ocular surface surgeries as well as in vitreoretinal surgeries. It has been used in ocular surface surgery for sealing corneal and conjunctival wound leaks, corneal stem cell transplantation, pterygium surgery, conjunctival closure after surgery, etc., (Pfister and Sommers, 2005; Srinivasan et al., 2009; Sonmez and Beden, 2011; Yang et al., 2013; Romano et al., 2016; Scalcione et al., 2018) Furthermore, isolated case reports have presented excellent results of fibrin glue as an adjunctive option in the surgical treatment of optic disc pit-associated macular detachment. (de Oliveira et al., 2017; Almeida et al., 2018) Therefore, it is theoretically feasible to apply fibrin glue to seal retinal breaks in patients with RRD based on favorable biocompatibility and sealing properties. Mudit et al. (Tyagi and Basu, 2019) reported that the initial clinical results of fibrin glue-assisted repair for RRD patients. Following the completion of PPV, fluid-air exchange, and laser photocoagulation around retinal breaks, the breaks were subsequently covered with 0.1–0.2 ml of fibrin glue (TISSEEL Kit, Baxter AG, Vienna, Austria), and subsequently the vitreous cavity was filled with the BSS after the glue formed a thick fibrin clot. At the end of the process, there was no evidence of glue migration or displacement and the patients were not instructed to maintain any specific postoperative body positioning. All the five cases (100%) had successful retinal reattachment, which was maintained after 3–8 months of follow-up. No eye showed any increase in postoperative inflammation or intraocular pressure (IOP), and electroretinography (ERG) revealed no remarkable alterations in retinal function. The milky-white color of the fibrin gel was visible during the follow-up period. The clump of fibrin glue significantly decreased in size after 1 week and completely dissolved after 2 weeks. However, the authors recommended avoiding using the dual-chambered syringe provided with the product during the fibrin glue application procedure because the glue tends to gel once there is contact between the two components (sealer protein and thrombin solutions) within the 25-gauge needle, which would block it. Preferably, the two components of the glue should be separately prepared in 1 ml insulin syringes. The procedure involves injecting a more viscous sealer protein solution to cover the retinal breaks completely, and then applying thrombin solution to mix them to form a gel. Likewise, the clinical trial conducted by Wang et al. (Wang et al., 2020) included a slightly larger sample size (26 eyes) and presented a 100% retinal reattachment rate during the entire follow-up period using the same surgical procedures as mentioned above. Commercially available fibrin gel was obtained from the Beixiu Porcine Fibrin Sealant Kit (Bei Xiu Biotech Co., Ltd., Guangzhou, China). Both pilot trials demonstrated that fibrin glue could be used as a substitute for endotamponades in certain RRD cases. Moreover, Erdinc et al. (Aydin et al., 2021) explored the use of fibrin glue as an alternative to laser photocoagulation for RRD treatment. After applying fibrin glue (TISSEEL Kit; Baxter AG, Vienna, Austria) on retinal breaks for 5 min, the vitreous cavity was filled with long-lasting vitreous substitutions. No laser photocoagulation was performed. All the five patients had successful retinal reattachment, and no postoperative complications were detected during the following 10–24 months.

These findings provide preliminary evidence to support the favorable biocompatibility and efficacy of commercially available fibrin glues as a prospective retinal patch for sealing retinal breaks in clinical RRD patients, and may potentially replace the use of endotamponades or laser photocoagulation. Nevertheless, all the clinical data available to date have been derived from small-scale clinical trials. More validation is needed, including longer follow-up, larger sample sizes, and multicenter clinical trials.

#### Gelatin-based sealants

Gelatin is a mixture of peptides and proteins obtained by the partial hydrolysis of collagen, and the peptide bonds link them together to form polymers with molecular weights ranging from 15,000 to 400,000 Da. (Echave et al., 2017) Gelatin has target sequences for the enzymatic degradation of matrix metalloproteinases (MMP), so the degradation products are biocompatible. (Backstrom and Tokes, 1995) The US Food and Drug Administration (FDA) has allowed the use of gelatin in biomedical applications because of its desirable qualities such as bioactive moieties, biocompatibility, biodegradability, and relatively low antigenicity. (Kang and Park, 2021) It is used clinically as a plasma expander and stabilizer in a variety of protein formulations, vaccinations, and gelatin sponges. (Elzoghby, 2013) However, owing to the relative instability of gelatin hydrogels in aqueous solutions (they swell and often dissolve above 35°C), several cross-linking methods, including chemical, photochemical, and enzymatic methods, have been utilized to provide stability under biological circumstances. (Scognamiglio et al., 2016; Echave et al., 2017) Traditionally, aldehydes are well-known chemical cross-linkers for peptides; however, they are hazardous to mammalian cells at high doses in the millimolar range because of the cytotoxicity, immunogenicity, and inflammatory effects of their degradation products. (Esterbauer et al., 1991) Therefore, there has been an increasing interest in using natural cross-linkers, such as transglutaminase or tyrosinase, to cross-link gelatin by enzymatic methods under physiological conditions. Nevertheless, direct cross-linking methods (without prior modification of gelatin) exhibit limited tailorability in the design of hydrogels, mainly because of poor control of the cross-link density and the resulting stiffness. Therefore, adding functional groups to the gelatin backbone before cross-linking provides a great degree of control over the design and properties of hydrogels. For these reasons, functionalized gelatin, followed by cross-linking, has overtaken direct cross-linking as the favored approach.

##### Enzymatically crosslinked gelatin (gelatin-mTG)

The gelatin microbial transglutaminase (gelatin-mTG) is generated by cross-linking gelatin molecules *via* the cross-linker of calcium-independent mTG enzyme, which covalent bond between a free amine group of a peptide-bound Lys and the acyl group at the end of the side chain of a peptide-bound Gln. (Scognamiglio et al., 2016) Gelatin-mTG could form a gel in 30 min under moist conditions, and this gel could withstand pressures of 200 mmHg in the *in vitro* test. The tensile static and dynamic loading of the adhesive hydrogels in bulk revealed that the Young’s modulus of gelatin-mTG ranged from 15 kPa to 120 kPa, and these bulk parameters were equivalent to those of fibrin-based sealant hydrogels. (McDermott et al., 2004; Liu et al., 2009) Therefore, based on the substantial adhesive and cohesive strength of gelatin-mTG, Chen et al. (Chen et al., 2006) first examined the potential of gelatin-mTG sealants (gelatin was obtained from Sigma Chemicals and mTG was purchased from Ajinomoto USA) for ophthalmic applications, especially for retinal reattachment. Gelatin-mTG sealants were prepared by mixing gelatin and mTG solutions for 1 min. The *in vitro* experiment demonstrated that the gelatin-mTG sealants could attach to moist retinal tissue, and the adhesive strengths achieved lap-shear strengths of 15–45 kPa over 30 min, which is comparable to strengths found with other soft-tissue adhesives. The *in vivo* histological study indicated that the components of gelatin-mTG are potentially biocompatible, since they did not induce cellular damage in the retinal tissues of rats after gelatin and mTG diffusion into the retina following vitreous cavity injection. Subsequently, Yamamoto et al. (Yamamoto et al., 2013) investigated the effects of using gelatin-mTG sealants for treating experimental RRD in rabbits. Reattachment of the retina occurred in all four eyes after retinal breaks were covered with gelatin-mTG sealants. Simultaneously, optical coherence tomography (OCT) revealed that the gelatin-mTG sealants were still adhered to the retina at least 1 week after the initial application, and ERG demonstrated that gelatin-mTG sealants had no negative impact on retinal function.

##### Photocrosslinked gelatin derivatives (GelMA)

Methacrylic anhydride (MA) is a well-known functional group that can partially substitute methacryloyl groups on reactive amines to obtain modified gelatin, that is, gelatin methacrylate (GelMA). GelMA is photoreactive and can be photocrosslinked *via* ultraviolet or visible light to achieve high physiological stability. In particular, the physical properties can be optimized for different applications by tuning the degree of gelatin modification, photoinitiator concentration, and cross-linking conditions. The chemical modification of gelatin by MA involves no more than 5% of the amino acid residues in the molar ratio, which implies that most of the functional amino acid domains of gelatin (such as MMP-degradable motifs) will be retained; thus, the GelMA hydrogels preserve the remarkable biocompatibility and bioactivity of gelatin. (Yue et al., 2015; Klotz et al., 2016) In terms of adhesion and sealing properties, GelMA outperforms clinically used fibrin glues and PEG-based glues *in vitro,* and is biocompatible enough to adequately seal large lung leaks and tracheal injuries in animal models. (Assmann et al., 2017; Gasek et al., 2021) Therefore, Chen et al. (Chen et al., 2021) engineered GelMA with varying degrees of substitution (25, 50, and 75%) and concentrations (10, 20, and 30%), and then tested and compared their adhesion, stability at 37°C, degradation time, swelling ratio, photo-crosslinking time, pH, and physical conditions before photo-crosslinking. In the *in vitro* study, the GelMA hydrogel with a 75% degree of substitution and a 20% concentration (75/20) displayed greater adhesion and stability than the other nine GelMA hydrogels (substitution/concentration) during the first 2 weeks. Furthermore, the GelMA hydrogel (75/20) was in gel condition at 37°C and could be injected with a 25-gauge needle before photo-crosslinking. The degradation time was reasonable, the swelling ratio was low, the photo-crosslinking time was short, and the pH was steady. Thus, the GelMA hydrogel (75/20) had the ideal formula. Histological examination showed that the retinal layer was not damaged 28 days after vitreous injection of the GelMA hydrogel (75/20). Although the animal RRD model has not been established to assess its effectiveness *in vivo*, preliminary conclusions could be drawn that GelMA (75/20) exhibits favorable intraocular biocompatibility, and it may be a suitable formulation for use as a patch to seal retinal breaks in RRD surgery.

In summary, gelatin-based sealants show great promise in preclinical *in vitro* and *in vivo* studies, indicating that they would be efficient with excellent prospects for clinical application. However, there are no clinical data regarding gelatin-based sealants for the treatment of RRD. Clearly, clinical trials in humans are necessary to completely evaluate the efficacy and safety of gelatin-based sealants for treating human RRD.

### Sealants based on polysaccharides

#### HA-based sealants

HA is a natural linear anionic polysaccharide with many distinct properties such as good biocompatibility and biodegradability, native biofunctionality, hydrophilicity, and non-immunoreactivity. (Ding et al., 2022) These properties contribute to its utilization as a promising candidate in clinical ophthalmology. HA has been used as an artificial tear ingredient to treat dry eyes, as well as a viscoelastic agent for cataract surgery. However, under natural conditions, numerous inherent limitations, including poor mechanical qualities and restricted cell adhesion, have hindered its wider utilization. These challenges can be solved by cross-linking HA chains to generate a gel-like structure that varies from its raw sol-like nature. Covalent cross-linking of HA can be achieved in two ways: by directly adding a cross-linker, or by first modifying the carboxyl, hydroxyl, or N-acetyl groups of HA chains with functional groups, followed by cross-linking. (Perez et al., 2021)

##### Seprafilm

Seprafilm is a translucent bioresorbable membrane composed of carboxymethylcellulose (CMC) and HA that has been chemically modified to slow the rate of degradation and clearance after implantation in the body. This membrane transforms the form of thin sheets into a hydrophilic gel and binds strongly to the target surface of tissues within 24–48 h after placement in a wet environment in the body. Animal experiments have shown that Seprafilm is absorbed from the application site within 7 days and is completely cleared within 28 days after implantation. Seprafilm is primarily used in abdominal and pelvic laparotomies asan adhesive physicochemical barrier to prevent adhesion between adjacent tissues and was approved by the FDA in 1996. (Diamond et al., 2012a; Diamond et al., 2012b; Guo et al., 2021) Afterwards, Seprafilm was utilized and investigated in an “off-label” capacity at the discretion of individual surgeons. Therefore, Tsurumaru et al. (Tsurumaru et al., 2009) investigated its efficacy in preventing postoperative adhesion between the conjunctiva and sclera during glaucoma filtering surgery. Animal studies have shown that it is effective in reducing adhesion and maintaining low eye pressure, and may be a desirable antifibrotic agent for trabeculectomy in the early stages of wound repair. Similarly, based on its adhesion and barrier properties, Jun et al. (Sueda et al., 2006) evaluated the safety and efficacy of Seprafilm (Genzyme Corporation, Cambridge, MA, USA) for sealing retinal breaks in animals. The *in vitro* investigation indicated that Seprafilm remained solid in BSS for 30 days before dissolving and that the pH of this solution was between 7.2 and 8.0, which is neutral for intraocular use. It also adhered well to the retina and effectively inhibited the permeation of methylene blue. After crushing and powdering the Seprafilm and mixing it with BSS, 0.1 ml of this solution was injected into the vitreous cavity of rabbits. This *in vivo* study demonstrated that there was no postoperative ocular inflammatory reaction during the 6-week follow-up, and ERG and histology proved that the morphology and function of the retina were normal before and after injection. As a result of technological advancements and these promising preliminary findings, Teruya et al. (Teruya et al., 2009) further investigated the short-term (14-day) effects of Seprafilm^®^ for patching retinal breaks in rabbit eyes with experimental RRD. The retina was reattached using Seprafilm as retinal patches during the 14-day observation period. Funduscopic examination and OCT revealed that the Seprafilm adhered tightly to the retina, the border of the breaks was indistinct on the 7th postoperative day, and Seprafilm dissolved within 14 days. Simultaneously, histological examination revealed that the Seprafilm application sites showed no inflammatory changes. Hence, the favorable biocompatibility and efficacy of seprifilm indicate that it is a potential retinal patch for repairing retinal breaks in clinical RRD cases. Subsequently, Haruta et al. (Haruta et al., 2017) confirmed the long-term safety of intraocular Seprafilm (Sanofi, Bridgewater, NJ, USA) in clinical RRD patients. Following laser photocoagulation around retinal breaks in four patients, a 5 × 2 mm sheet of Seprafilm was placed over the retinal breaks after being delivered into the eye through one of the sclerotomy sites that had been enlarged to 3 mm. The retina remained reattached in all the four eyes at 1, 3, and 6 months and 9 years postoperatively, with no immediate or late adverse events. Electroretinography and specular microscopy revealed no evidence of Seprafilm toxicity 9 years after the surgery.

##### Healaflow

Healaflow (Anteis S.A., Plan Les Ouates, Switzerland) is a commercially available translucent hydrogel made of over 97% water and reticulated HA of non-animal origin (22.5 mg/ml) using 1,4-butanediol diglycidyl ether (BDDE) as a cross-linker. BDDE is a well-known industry-standard cross-linker of market-leading cross-linked HA products. Its stability and metabolism have been thoroughly examined, and long-term safety has been confirmed by various studies and clinical trials over the years. (De Boulle et al., 2013) Healaflow is initially used in glaucoma filtration procedures as a space-filler to minimize postoperative fibrosis, and it usually takes about 3 months for the body to completely degrade. Its effect on maintaining functional filtration blebs and controlling and stabilizing intraocular pressure is comparable to that of mitomycin-C. (Bettin et al., 2016; Mudhol and Bansal, 2021; Wu et al., 2021) The composition of Healaflow is similar to that of natural vitreous: a reinforced hydrogel composed of HA with similar light transmittance as well as physical and mechanical properties. Therefore, it is a plausible candidate for vitreous substitution. Accordingly, Barth et al. (Barth et al., 2014) introduced the retinal explant assay as a valuable technique for early biocompatibility assessment, prior to more expensive and time-consuming *in vivo* testing. The neurosensory retina of the rat eyes was dissected from the RPE and vitreous, explanted onto culture plate inserts, and co-cultured after being covered with hydrogel. In this setting, retinal explants cultured with Healaflow outperformed standard-cultured specimens and appeared to reduce the trauma induced by the culture process. Further *in vivo* biocompatibility testing was conducted. After central vitrectomy and fluid-air exchange, approximately 0.5–1 ml Healaflow was injected into the vitreous cavity *via* a 25-gauge needle. Postoperative clinical evaluation, ERG, histology, and immunohistochemistry clearly showed that Healaflow displayed good biocompatibility with no morphological or functional alterations to the retina. (Barth et al., 2016; Barth et al., 2019) Ren et al. (Ren et al., 2020) described the *in vitro* testing of Healaflow adhesion. That is, Healaflow was applied to three walls of a culture flask filled with BSS (the inside surface of the lid, side wall, and bottom), and 14 days later, Healaflow remained adherent on the three walls with no change in size. On the premise that Healaflow combines good biocompatibility and adhesion, the authors further explored the potential of Healaflow as a retinal patch for the treatment of clinical primary RRD. After complete vitrectomy, fluid-air exchange, and laser photocoagulation around the retinal breaks, Healaflow was applied to the surface of all breaks with a 27-gauge needle in 37 patients (38 eyes). Primary reattachment was achieved in 37 eyes (97.4%) due to failure of the chorioretinal scar around the retinal breaks in one eye. Final reattachment was achieved in all eyes (100%) after the second surgery, with no significant adverse complications.

To summarize, these two commercially available HA-based sealants were tested in preclinical *in vitro* and *in vivo* experiments, as well as in clinical RRD patients, and showed good efficacy and safety as retinal patches for RRD treatment. HA-based sealants sealing retinal breaks could replace long-standing vitreous substitutes, allowing for earlier visual recovery and fewer complications. However, unlike fibrin glue, neither of the two HA-based sealants displayed alternative properties to laser photocoagulation. Ren et al. (Ren et al., 2020) described a patient with failed retinal reattachment due to the lack of chorioretinal scarring around retinal breaks. However, Healaflow is better than Seprifilm in terms of flexibility. The sheet shape of Seprafilm necessitates expansion of the sclerotomy sites to deliver it into the eye, and the conditions must be kept moist to transform it into a hydrophilic gel. In contrast, the gel nature and injectable properties of Healaflow make it easy to deliver into the eye and cover retinal breaks.

#### Chondroitin sulfate-based sealants

Ophthalmic viscosurgical devices (OVDs) with both solid and fluid properties are commonly used in anterior segment surgery, such as in challenging cataracts, flat anterior chambers, pseudoexfoliation syndrome, intraoperative floppy iris syndrome, or glaucoma surgery. Its main physical properties not only create and maintain anterior chamber depth and visibility but also protect corneal endothelial cells and other intraocular tissues. (Bissen-Miyajima, 2008; Borkenstein et al., 2021) The commonly used OVDs are composed of the following three building blocks: sodium HA, chondroitin sulfate, and hydroxypropyl methylcellulose. Chondroitin sulfate is a sulfated glycosaminoglycan that is composed of alternating sugar chains. It is often associated with proteins as a component of proteoglycans. OVDs containing chondroitin sulfate are known as dispersive OVDs, which have a stronger adhesive ability than other cohesive OVDs. (Hirata et al., 2013) Hirata et al. (Hirata et al., 2013) investigated the efficiency of retinal reattachment in four experimental rabbit RRD eyes by short-term patching of retinal breaks with a dispersive OVD (Viscoat, Alcon, Japan), based on the premise of its strong adhesive ability. After temporarily flattening the detached retina and performing laser photocoagulation around the retinal breaks, then the dispersive OVD was injected on the retinal surface to patch retinal breaks. Postoperative inflammation did not differ between rabbit eyes received dispersive OVD as a retinal patch and those who did not (the control group). OCT revealed that the dispersive OVD was slightly decreased, however, it was still adequate to patch the retinal break 3 days after surgery. Seven days later, the dispersive OVD disappeared, the retina was repositioned, and no re-detachment was observed. Alternatively, retinal detachment persisted in the control group. Furthermore, scanning electron microscopy demonstrated that in the control group, the edge of the retinal breaks was clearly identifiable and the surrounding retina was floating, while in the eyes with dispersive OVD, the breaks were indistinct and the exposed areas were covered with glial cells. Measurements showed a significant reduction in the exposed RPE area in the dispersed OVD group.

These results suggest that OVD is a promising new treatment option for RRD, however, further clinical trials are needed to verify its effectiveness and safety.

## Sealants of synthetic materials

The performance inefficacy, safety issues (immune responses, viral transmission, possible cytotoxicity triggered by residual chemical cross-linkers, etc.), and limitations related to the application of some natural-based surgical sealants have pushed researchers to develop synthetic sealants, whose precise control over their mechanical properties *via* modification of a variety of functional groups makes them perform better in the biological environment, and have more application areas and fewer drawbacks. Synthetic adhesives are composed of synthetic chemicals, typically monomers, pre-polymers, or non-cross-linked polymers, which undergo *in situ* polymerization or cross-linking to form insoluble adhesive matrices when applied to tissues. (Mehdizadeh and Yang, 2013) Various types of synthetic sealant materials have been used for general surgical applications. Generally, synthetic sealant materials fall into one of the three categories: cyanoacrylates, PEGs, or polyurethane. However, in terms of retinal patches for RRD treatment, there are no studies on polyurethane-based sealants. Meanwhile, after the localized but definite retinal toxicity was validated by histological examinations in rabbit eyes 1 month after cyanoacrylate implantation, (Hida et al., 1988) further clinical studies were prevented. Currently, the synthetic sealants used as retinal patches for the treatment of RRD are PEG-based sealants.

### PEG-based sealants

PEG is a synthetic, inert, water-soluble polymer that is widely used in the biomedical field owing to its excellent biocompatibility and non-immunogenicity. Modification by degradable functionalities or copolymerization with biodegradable polymers can mask the lack of biodegradability, which enables its successful use in a variety of biomedical applications, such as acting as a hemostat or a fluid barrier *via* different end groups. (Scognamiglio et al., 2016) For example, the PEG hydrogel (DuraSeal Dural Sealant System) has been approved by the US FDA for obtaining watertight dural closure when applied after standard dural suturing. (Osbun et al., 2012) In addition, in the field of ophthalmology, the PEG-based synthetic sealant could also act as an adhesion barrier to prevent leakage of filtration blebs and as an adhesive to close sclerotomies of PPV in rabbit eyes. (Hoshi et al., 2016; Nagata et al., 2020).

In preclinical studies of RRD, PEG-based sealants from different brands showed excellent biocompatibility and efficacy in both *in vitro* and *in vivo* investigations when applied as retinal patches to close retinal breaks for the treatment of RRD. Table 4 summarizes the studies on different brands. However, no clinical data are available to date. The brand of DuraSeal^®^ (Confluent Surgical, Waltham, MA, USA) is composed of two synthetic liquids: a PEG solution with a gel-like consistency (>90% water) and an amine component that serves as a precursor. Polymerization occurred within 20 s of mixing the two components without heat formation. (Sueda et al., 2007; Barliya et al., 2018) The brand of Medicus polymer (Medicus Biosciences, San José, California, USA) comprised solutions of PEG 8-arm acetate amine combined with solutions of PEGylated 8-arm ester dissolved in physiological buffer solution and a viscosity enhancer. In mature market commodities, the component is kept in powder form in a syringe and must be combined with a viscosity enhancer (methylcellulose) before being used in hydrogel conditions. (Kerr, 2014; Sarfare et al., 2015; Hubschman et al., 2017) One thing that makes this brand of compound unique compared to other PEG-based sealants is that the kinetics of its degradation polymerization can be customized by the surgeons themselves *via* different formulations. (Hubschman et al., 2017) The brand of FocalSeal^®^ (Genzyme Corporation, Cambridge, MA, USA) is an ethylene glycol-oligotrimethylene carbonate copolymer that has been end-capped with acrylate esters. Triethanolamine (90 mM) and eosin Y were also present in the solution, acting as photoinitiators. A transparent, flexible, and securely adhering hydrogel was created by polymerizing the solution for 40–60 s with visible illumination from a xenon arc lamp (450–500 nm, blue-green). (Hoshi et al., 2015; Hoshi et al., 2018).

**TABLE 4:** The summaries of studies about PEG-based sealants as a retinal patch for the treatment of RRD.

PED-based sealants	Authors	Date	Preclinical studies	Clinical trails
DuraSeal® (Confluent Surgical, Waltham, MA, USA)	Sueda (Sueda et al., 2007)	2007	*In vitro* study about polymerization and degradation time.	None
			*In vivo* study about biocompatibility in rabbit eyes.	
			*In vivo* study about efficacy in RRD model of rabbit eyes.	
DuraSeal® (Confluent Surgical, Waltham, MA, USA)	Barliya (Barliya et al., 2018)	2018	*In vivo* study about biocompatibility and efficacy in RRD model of rabbit eyes. Differently, the two components of the hydrogel mix and polymerize at subretinal space and then close the retinal breaks.	None
Medicus polymer (Medicus Biosciences, San José, California, USA)	Kerr (Kerr, 2014)	2014	*In vitro* study about biomechanics.	None
Medicus polymer (Medicus Biosciences, San José, California, USA)	Sarfare (Sarfare et al., 2015)	2015	*In vivo* study about biocompatibility in mice eyes.	None
Medicus polymer (Medicus Biosciences, San José, California, USA)	Hubschman (Hubschman et al., 2017)	2017	*In vivo* study about biocompatibility and efficacy in RRD model of pig eyes.	None
FocalSeal® (Genzyme Corporation, Cambridge, MA, USA)	Hosin (Hoshi et al., 2015)	2015	*In vitro* study about adhesion.	None
			*In vivo* study about biocompatibility in rabbit eyes.	
FocalSeal® (Genzyme Corporation, Cambridge, MA, USA)	Hosin (Hoshi et al., 2018)	2018	*In vivo* study about biocompatibility and efficacy in RRD model of rabbit eyes.	None

RRD, rhegmatogenous retinal detachment.

Similar to the safety and efficacy reports of PEG hydrogel as a dural sealant in multicenter, single-blind, prospective randomized cranial surgery, (Osbun et al., 2012) it is believed that multicenter and large-scale clinical trials on PEG-based sealants as retinal patches will also be reported shortly.

## Outlook

In the natural biological world, a range of species, such as mussels and barnacles, secrete substances that assist them in underwater attachment. The strong adhesion of mussels to a surface is based on the mussel adhesive protein (MAP), in which 3,4 dihydroxy-L-phenyalanine (DOPA) has been identified as the key factor that can penetrate water boundary layers and subsequently bond strongly with surfaces. (Zhang et al., 2020) The MAP model is well known for its promising adhesive performance to wet surfaces; therefore, mussel-inspired adhesives have been designed and tested in preclinical studies. (Lee et al., 2011; Rahimnejad and Zhong, 2017; Kianersi et al., 2020; Pandey et al., 2020) Now the research field is moving towards the use of nanoparticles to create next-generation mussel-inspired adhesives with increased adhesive strength and drug delivery for tissue regeneration. A low concentration of MAP is biocompatible with the retina. (Liggett et al., 1990) Moreover, magnetic nanoparticle bioadhesive compounds can effectively seal retinal breaks in rabbit eyes. (Ji et al., 2018) Although there has been a surge in research on mussel-inspired adhesives in recent years, none of these adhesives have been reported in clinical trials. However, they have received great interest from the research community. Therefore, mussel-inspired adhesives may be another potential option for retinal patches in the treatment of RRD.

## Conclusion

The progress of technology has made biomaterials progress from simple original material to the current cross-linked or polymerized materials, and from single modification to the coexistence of multiple modifications. These advances have reduced the retinal damage caused by the toxicity of biomaterials, difficulties in delivery, as well as enhanced biocompatibility and efficacy, while simultaneously making significant contributions to the treatment alteration of RRD, quick recovery of vision, and avoidance of postoperative face-down position. Although research on biomaterials as retinal patches for RRD repair is in full swing, most are in the primary stage of research, and few materials have entered clinical applications. There is still a long way to go and carry further exploration before biomaterials become a standard procedure. The challenges in the translation to clinical studies are summarized as follows. First, in experimental eyes with RRD, small round retinal breaks are usually generated at the inferior region, resulting in a localized retinal detachment that lasts only a few seconds. Conversely, the location and morphology of retinal breaks in patients with RRD remain uncertain. Importantly, retinal detachment lasts significantly longer and has more microenvironmental alterations; hence, experimental RRD could not fully imitate the pathological processes of clinical RRD. Therefore, some patients and ophthalmologists are disinclined to undergo initial trials based on the preclinical outcomes. Second, the precise application of sealants over retinal breaks is challenging. Visualization through air is difficult for transparent sealants, such as the clinically used Healaflow. In addition, there is a risk of sealant migration to the subretinal space owing to fluidity, such as the two solution components of fibrin glue. Additionally, if there are remaining retinal folds across which the sealant flows, it might result in retino-retinal adhesions. For superior retinal breaks, it is difficult to attach sealants successfully because of the spherical shape of the eyeball and the effect of gravity; especially the sealant requires a few seconds to gel, such as the clinically used fibrin glue, whose two components need to be applied one by one. Therefore, a learning curve exists. Third, the indication of biomaterials as retinal patches overlaps with that of SB; therefore, some patients and ophthalmologists prefer SB with a long history and no difference in the success rate with PPV, which would limit the application and dissemination of biomaterials as retinal patches. Finally, the cost of sealants must be considered in terms of the surgical cost. All of these factors contribute to the fact that, despite encouraging results, some sealants are currently only available for preclinical testing, or that, while the remaining sealants have undergone clinical trials, all clinical studies are on small scales, and the condition of RRD patients is simple. To date, whether these biomaterials as retinal patches can completely replace long-lasting vitreous substitutions in cases of complex RRD has not been reported. Therefore, more randomized, prospective, large-scale clinical trials of biomaterials as retinal patches need to be conducted to definitively determine their biocompatibility, efficacy, and potential for complete replacement of long-lasting vitreous substitutes in RRD patients.
